# Long non-coding RNA ZEB1-AS1 regulates miR-200b/FSCN1 signaling and enhances migration and invasion induced by TGF-β1 in bladder cancer cells

**DOI:** 10.1186/s13046-019-1102-6

**Published:** 2019-03-01

**Authors:** Ruxu Gao, Naiwen Zhang, Jianyu Yang, Yuyan Zhu, Zhe Zhang, Jianfeng Wang, Xiaolong Xu, Zeliang Li, Xiankui Liu, Zhenhua Li, Jun Li, Chuize Kong, Jianbin Bi

**Affiliations:** Department of Urology, The First Hospital of China Medical University, Shenyang, Liaoning 110001 People’s Republic of China

**Keywords:** Bladder cancer, fascin1, Long non-coding RNA *ZEB1-AS1*, microRNA miR-200b, TGF-β1

## Abstract

**Background:**

The effect of competing endogenous RNA (ceRNA) can regulate gene expression by competitively binding microRNAs. Fascin-1 (FSCN1) plays an important role in the regulation of cellular migration and invasion during tumor progression, but how its regulatory mechanism works through the ceRNA effect is still unclear in bladder cancer (BLCA).

**Methods:**

The role of fascin-1, *miR-200b*, and *ZEB1-AS1* in BLCA was investigated in vitro and in vivo. The interaction between fascin-1, *miR-200b*, and *ZEB1-AS1* was identified using bioinformatics analysis, luciferase activity assays, RNA-binding protein immunoprecipitation (RIP), quantitative PCR, and western blotting. Loss (or gain)-of-function experiments were performed to investigate the biological roles of *miR-200b* and *ZEB1-AS1* on migration, invasion, proliferation, cell apoptosis, and cell cycle.

**Results:**

*ZEB1-AS1* functions as a competing endogenous RNA in BLCA to regulate the expression of fascin-1 through *miR-200b*. Moreover, the oncogenic long non-coding RNA *ZEB1-AS1* was highly expressed in BLCA and positively correlated with high tumor grade, high TNM stage, and reduced survival of patients with BLCA. Moreover, *ZEB1-AS1* downregulated the expression of *miR-200b*, promoted migration, invasion, and proliferation, and inhibited apoptosis in BLCA. Furthermore, we found TGF-β1 induced migration and invasion in BLCA by regulating the *ZEB1-AS1*/*miR-200b*/FSCN1 axis.

**Conclusion:**

The observations in this study identify an important regulatory mechanism of fascin-1 in BLCA, and the TGF-β1/*ZEB1-AS1*/*miR-200b*/FSCN1 axis may serve as a potential target for cancer therapeutic purposes.

**Electronic supplementary material:**

The online version of this article (10.1186/s13046-019-1102-6) contains supplementary material, which is available to authorized users.

## Background

Bladder cancer (BLCA) is the most common urinary cancer worldwide [1]. Several genes have been associated with BLCA tumorigenesis. However, the molecular mechanisms underlying the progression of BLCA are still unclear.

Fascin-1 (FSCN1) is a globular actin-bundling protein [2]. There are three isoforms of fascin in vertebrate cells. Among them, FSCN1 is expressed in mesenchymal tissues and the nervous system, FSCN2 is expressed in the retinal photoreceptors, and FSCN3 is a testis-specific protein [3]. FSCN1 is located at the membrane ruffles, micro spikes, and stress fibers and induces membrane protuberance. The main function of FSCN1 is the formation of parallel actin bundles, which support filopodial and lamellipodial cell protrusions that are key cellular structures related to environmental guidance and cell movement, migration, and adhesion [4–6].

Recent studies have shown that FSCN1 levels are significantly increased in transformed epithelial cells as well as in various types of carcinomas [7–9], including BLCA [10, 11]. Our previous studies have found that FSCN1 promoted migration and invasion of BLCA [12, 13].

In the present study, through bioinformatic analysis, we found that the microRNA (miRNA) *miR-200b* may regulate *FSCN1* expression. *miR-200b* has been shown to be a tumor suppressor in multiple cancer types, including BLCA. However, the expression pattern of *miR-200b* in BLCA is intriguing, in that it is higher in BLCA tissues than in normal bladder tissues, but lower in high grade tumors than in low grade tumors [14].

Long non-coding RNAs (lncRNAs) have been the focus of numerous studies in recent years. It has been suggested that lncRNAs act as “sponges” for microRNAs, reducing their effect on mRNAs and therefore regulating several biological processes. In the present study, we found that the lncRNA *ZEB1-AS1* may regulate *miR-200b*. Zinc Finger E-Box Binding Homeobox 1 (ZEB1) is a promoter of epithelial-to-mesenchymal transition (EMT) and a target of *miR-200b*. Additionally, lncRNA *ZEB1-AS1*, which is the antisense of ZEB1, has been shown to be an oncogenic lncRNA in many kinds of cancers [15].

Transforming growth factor (TGF)-β has been shown to be an oncogene that can affect several signaling pathways in cancer cells [16, 17]. It has been previously reported that TGF-β1 downregulates the expression of *miR-200 s* [18] and upregulates the expression of *FSCN1* [19]. However, the molecular details underlying this process are still unclear. In the present study, we found that *ZEB1-AS1* is a downstream target of TGF-β1 and is involved in its regulatory mechanism on cell migration and invasion by affecting *miR-200b*/FSCN1 axis in BLCA cells. Our data might add insight on the diagnosis and treatment of BLCA.

## Methods

### Human samples

Ten paired BLCA and corresponding noncancerous tissues (located > 3 cm from the tumor), 60 BLCA tissue samples, and 23 normal tissue samples were obtained from patients at the Department of Urology, The First Hospital of China Medical University, between 2012 and 2017. The protocols used in the study were approved by the Hospital’s Protection of Human Subjects Committee.

### Cell culture and transfection

The human bladder carcinoma cell line RT4 was cultured in McCoy5A medium (HyClone, Logan, UT, USA), while the cell lines SW780, BIU, 5637, J82, T24, TCC-SUP, UM-UC3 were cultured in RPMI 1640 (HyClone). McCoy5A and RPMI 1640 were supplemented with 10% fetal bovine serum (FBS; HyClone), and the cells were cultured in a humidified atmosphere with 5% CO_2_ at 37 °C. Lipofectamine™ 3000 (Invitrogen, Carlsbad, CA, USA) was used for transfections, according to the manufacturer’s recommendations.

### Plasmids and short interfering RNA (siRNA)

Human pcDNA3.1-*ZEB1-AS1* plasmid, pcDNA3.1-negative control (NC), siRNA against *ZEB1-AS1* (siZEB1-AS1), siRNA against *FSCN1* (siFSCN1), hsa-mir-200b-3p mimics (miR-200b), mimics NC (miR-NC), hsa-mir-200b-3p inhibitor (ant miR-200b), inhibitor NC (ant miR-NC), and the pmirGLO luciferase reporter plasmid were synthesized by and purchased from GenePharm (Shanghai, China). RNAi sequences are shown in Additional file 1: Table S1.

### Dual luciferase reporter assay

Cells were seeded (4 × 10^4^ cells/well) in triplicate in 24-well plates and cultured for 24 h. RNA/DNA was transfected according to the experimental purpose. Luciferase and Renilla signals were measured 48 h after treatment using a Dual Luciferase Reporter Assay Kit (Promega, Madison, WI, USA) according to the manufacturer’s protocol.

### RNA extraction and quantitative PCR (qPCR)

Total RNA (including miRNA) from cells and bladder tissues was extracted using the miRNeasy™ Mini Kit (Qiagen, Hilden, Germany) according to the manufacturer’s recommendations. Nuclear RNA from cells was extracted with the miRNeasy™ Mini Kit after nuclear extraction with a Nuclear Extraction Kit (Solarbio, Beijing, China).

cDNA (except for cDNA from miRNA) was synthesized with the PrimeScript™ RT Master Mix (Takara, Beijing, China). cDNA of miRNA was synthesized using the Mir-X™ miRNA First-Strand Synthesis Kit (Clontech Laboratories). qPCR was performed using the SYBR Premix EX Taq™ (Takara). The 2^-ΔΔCT^ method was used to calculate the relative expression level. Primer pairs used for qPCR are shown in Additional file 1: Table S2.

### Western blotting

Cells were lysed in radioimmunoprecipitation assay (RIPA) buffer. Protein concentrations were detected using a bicinchoninic acid (BCA) assay kit. Equal amounts of protein samples were separated by 10% sodium dodecyl sulphate-polyacrylamide gel electrophoresis and then transferred to polyvinylidene fluoride membranes. The membranes were blocked with 5% skim milk in Tris-buffered saline with 1% Tween 20 (TBS-T) for 1 h and then incubated with the appropriate primary antibodies at 4 °C overnight. After washing with TBS-T, the membranes were incubated with horseradish peroxidase-conjugated secondary antibodies at 37 °C for 1 h. The membranes were then washed and the enhanced chemiluminescence method was used for protein detection according to the manufacturer’s instructions. Antibodies against FSCN1, E-cadherin and N-cadherin were purchased from Abcam (Cambridge, MA, USA). The antibody against vimentin was purchased from Santa Cruz Biotechnology (Dallas, TX, USA). The antibody against glyceraldehyde 3-phosphate dehydrogenase (GAPDH; loading control) was purchased from Sigma-Aldrich (St. Louis, MO, USA).

### Transwell assays

Cell invasion and migration were measured using transwell chambers with 8-μm pores in 24-well tissue culture plates (Corning Costar, Corning, NY, USA). Transwell chambers coated with Matrigel (BD, San Diego, CA, USA) were used to determine cell invasiveness, while transwell chambers without Matrigel were used to measure cell motility. After transfection of DNA/RNA for 48 h, the cells were re-suspended in RPMI 1640 containing 1% FBS, and 0.2 mL cell suspension (1 × 10^4^ cells per well for cell migration, 2 × 10^4^ cells per well for cell invasion) was seeded into the top chamber, whereas the lower chamber was filled with 0.6 ml per well of RPMI 1640 containing 10% FBS, used as a chemoattractant.

After 24 h of incubation at 37 °C, the cells that remained on the upper side of the filter were removed using cotton swabs, and those that had migrated to the lower side were fixed and stained with 1.0% crystal violet. Images were captured (100 ×) by microscope, and cells were counted using the ImageJ 1.48v software (National institutes of health, Bethesda, Maryland, USA).

### Cell cycle analysis

Cells were trypsinized and washed in ice-cold phosphate-buffered saline (PBS) and then fixed in ice-cold 75% ethanol in PBS. A propidium iodide (PI)/RNase staining buffer (BD) was added, and the cells were incubated at 4 °C for 30 min. Cell cycle profiles were analyzed using a FACS Calibur flow cytometer (BD).

### Cell apoptosis assays

Cells were detached using trypsin without ethylenediaminetetraacetic acid (EDTA). An Annexin V- fluorescein isothiocyanate (FITC)/PI (BD) solution was added, and the cells were incubated for 15 min at room temperature. Cell apoptosis were analyzed using the FACS Calibur.

### 5-Ethynyl-2′-deoxyuridine (EdU)

T24 and RT4 cells were seeded in 96-well plates at a density of 5000 cells per well and cultured for 24 h. The solution of an EdU Kit (Ribobio, Guangzhou, China) was diluted in proportion to 1: 1000 in cell medium. Cells were incubated with the EdU solution for 2 h and were stained following the manufacturer’s instructions. Images were acquired using a fluorescence microscope.

### Real time cell analysis (RTCA)

T24 and RT4 cells were seeded in E-plates at a density of 5000 cells per well and incubated in culture medium at 37 °C in a 5% CO_2_ atmosphere for 72 h. Cell growth curves were automatically recorded by the RTCA xCELLigence S16.

### Fluorescence in situ hybridization (FISH)

The probe of ZEB1-AS1 was designed and purchased from GenePharm (Shanghai, China).

Sequences of the ZEB1-AS1 probe are shown in Additional file 1: Table S3. T24 and RT4 cells were seeded in 48-well plates at a density of 10,000 cells per well and incubated in culture medium at 37 °C in a 5% CO_2_ atmosphere overnight. Next, the cells were washed twice with PBS, fixed with 4% paraformaldehyde for 15 min. As per the manufacturer’s instructions, the plate was placed at 73 °C for 5 min and incubated at 37 °C for 12~16 h in an incubator after adding the mixture of 100 μl (10 μM) probes. Then, the cells were stained with 200 μl (500 ng/ml) of 4′, 6-diamidino-2-phenylindole (DAPI; Beyotime, Shanghai, China) per well in the dark for 1 min after being washed following the manufacturer’s instructions. Images were acquired using a fluorescence microscope.

### RNA-binding protein immunoprecipitation (RIP)

The Magna RIP kit (Millipore) was used for RIP assays with the anti-Ago2 antibody (Abcam), according to the manufacturer’s recommendations.

### Phalloidin staining

T24 cells were seeded in 24-well plates. When the cell density reached 50%, the cells were washed twice with PBS, fixed with 4% paraformaldehyde and permeabilized with 0.5% Triton-X-100. Next, the cells were incubated with 200 μl (5 μg/ml) Rhodamine Phalloidin (YEASEN, Shanghai, China) per well in the dark for 30 min and then strained with 200 μl (500 ng/ml) of 4′, 6-diamidino-2-phenylindole (DAPI; Beyotime, Shanghai, China) per well in the dark for 1 min. Images were acquired using a fluorescence microscope.

### Xenograft tumor model

BALB/c nude mice (4–6 weeks old, 14–16 g) were purchased from Beijing Vital River Experimental Animal Technology Co, Ltd. (Beijing, China). The mice were housed in barrier facilities on a 12-h light/dark cycle. The Institutional Animal Care and Use Committee of China Medical University approved all the experimental procedures. Twelve mice were inoculated subcutaneously with UM-UC3 cells (5 × 10^6^) in the right dorsal flanks. The mice were randomly divided into two groups (*n* = 6/group), injected with UM-UC3 cells transfected with pcDNA3.1-*ZEB1-AS1* or pcDNA3.1-NC. Tumors were examined twice weekly; their length, width, and thickness were measured with calipers, and tumor volumes were calculated using the equation (Length× Width^2^)/2. On day 60, the animals were euthanized, and the tumors were excised, weighed, and paraffin-embedded. Serial 6.0-μm sections were cut and subjected to immunohistochemistry (IHC). The Ki67 and FSCN1 antibodies were used in these assays.

### TGF-β treatment

TGF-β (R&D Systems) was stored at − 20 °C after reconstitution in sterile ddH_2_O. When needed, TGF-β was diluted in serum-free medium to a concentration of 10 ng/ml and added to the cells.

### Statistical analysis

All data were expressed as the mean ± standard deviation (SD), and represented as the average of at least three experiments, with each experiment performed in triplicate. Statistical significance was determined using the Student’s t-test (two-tailed) or Mann-Whitney U test. The correlation between expression of different genes was evaluated by Spearman correlation analysis. The correlation between patients’ clinical pathological characteristics and *ZEB1-AS1* expression were analyzed by the Chi-square test. In bar graphs, * and ** indicate *P* < 0.05 and *P* < 0.01, respectively. A *P* value < 0.05 was considered to indicate statistical significance.

## Results

### The expression of *FSCN1* is negatively correlated with that of *miR-200b* in BLCA tissues and cells

Firstly, we searched for miRNAs that could potentially regulate *FSCN1* expression using the Targetscan server (http://www.targetscan.org) [20] and found that *miR-200b* was a valid candidate on the basis of the potential *miR-200b* binding site on *FSCN1* mRNA.

**Fig. 1:**
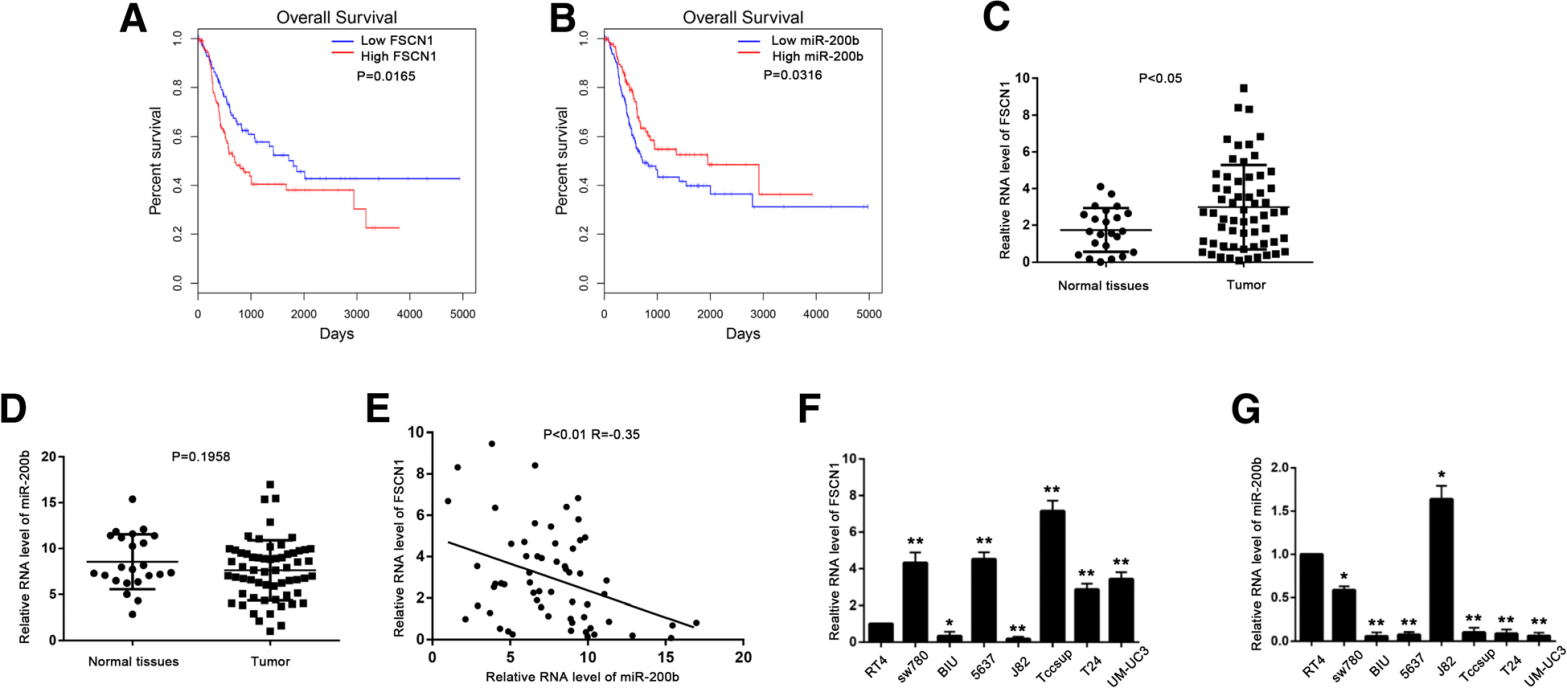
Differential expression of fascin-1 (*FSCN1*) and *miR-200b* in bladder cancer (BLCA) tissues and cells. **a** and **b** Kaplan–Meier analysis of overall survival based on *FSCN1* (**a**) and *miR-200b* (**b**) expression levels in patients with BLCA from the TCGA database. Patients with BLCA were divided into *FSCN1* (or *miR-200b*) high expression group (33% of the total, with the highest *FSCN1* (or *miR-200b*) expression) and low expression group (33%, with the lowest *FSCN1* (or *miR-200b*) expression). **c** and **d** The mRNA level of *FSCN1* (**c**) and *miR-200b* (**d**) in 23 normal bladder tissues and 60 BLCA tissues was measured by qPCR. Data were analyzed by Mann-Whitney U test. **e** Correlation between *FSCN1* and *miR-200b* of 60 BLCA tissues. Data were analyzed by Spearman correlation analysis. **f** and **g** The mRNA level of *FSCN1* (**f**) and *miR-200b* (**g**) in BLCA cell lines was measured by quantitative PCR (qPCR) and normalized by RT4 cell line. Data were analyzed by T-test. Data are presented as the mean ± standard deviation (SD). *P < 0.05; **P < 0.01; ***P < 0.001; ns, not significant

According to OncoLnc (www.oncolnc.org; a TCGA data portal) [21], high levels of *FSCN1* indicated poor prognosis (Fig. 1a); conversely, high *miR-200b* expression indicated good prognosis (Fig. 1b). Additionally, *FSCN1* expression was negatively correlated with *miR-200b* in BLCA (Additional file 2: Figure S1A). Next, we detected *FSCN1* and *miR-200b* levels in 23 normal bladder samples and 60 BLCA tissues. We found that *FSCN1* was upregulated (Fig. 1c) in cancer tissues, but there was no significant difference in *miR-200b* expression between BLCA tissues and normal bladder tissues (Fig. 1d). However, in the BLCA tissues, the expression of *FSCN1* was negatively correlated with that of *miR-200b* (Fig. 1e). We then detected the mRNA levels of *FSCN1* and *miR-200b* in BLCA cell lines and found that *FSCN1* was highly expressed in some high grade BLCA cell lines (Tccsup, T24, UM-UC3), in which the expression level of *miR-200b* was low (Fig. 1f and g).

These results suggest that the expression of *FSCN1* is negatively correlated with that of *miR-200b* in BLCA tissues and cells.

### *miR-200b* is a potential suppressor for *FSCN1*

**Fig. 2:**
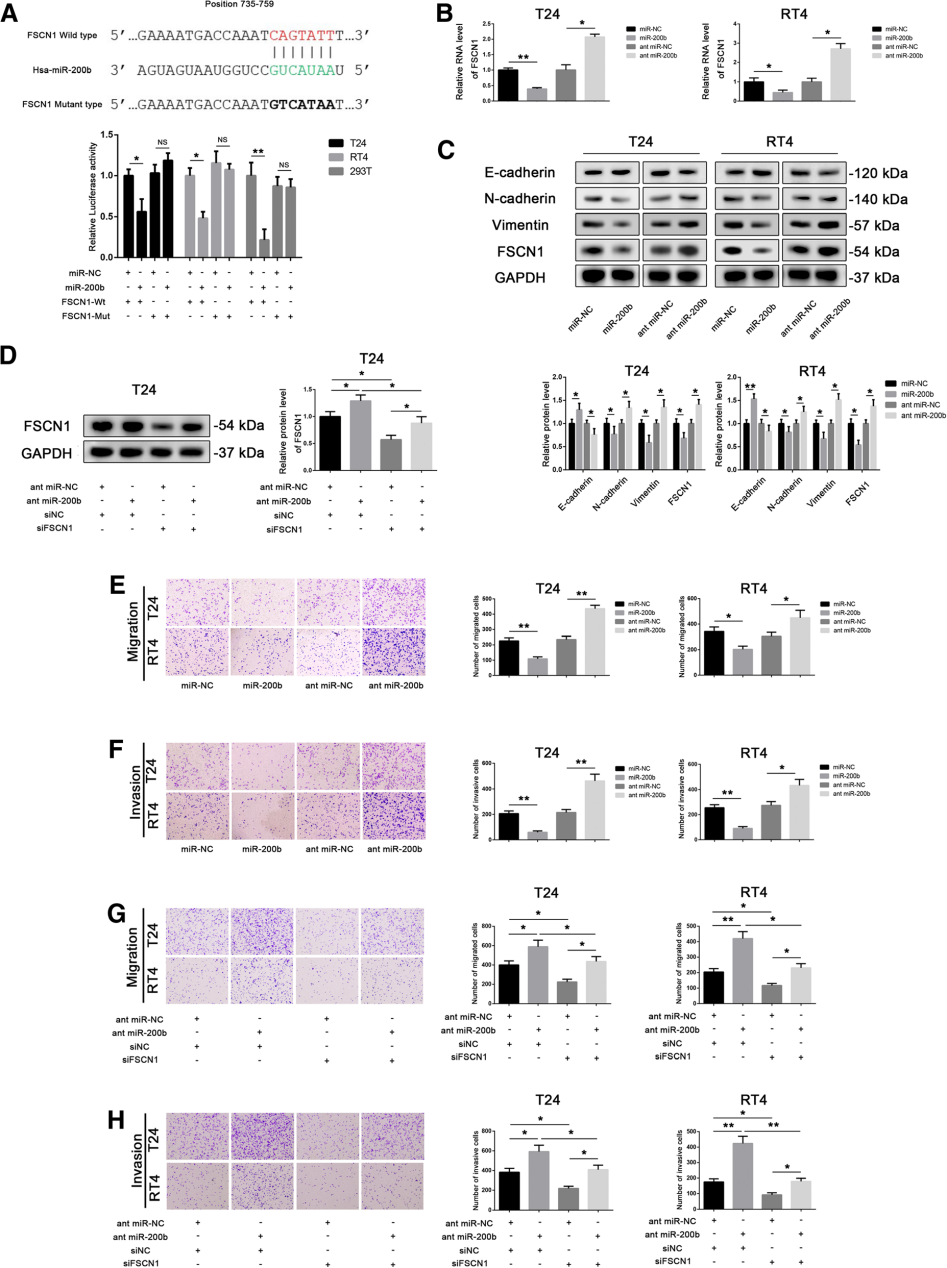
*miR-200b* inhibits fascin-1 (*FSCN1*) expression, migration, invasion. **a** Luciferase assays were performed after T24, RT4 and 293 T cells were co-transfected for 24 h with miR-negative control (*miR-NC*) or *miR-200b* and a plasmid containing wild-type or mutant-type *FSCN1* 3′ untranslated region (UTR) upstream the luciferase gene. The firefly luciferase activity of each sample was normalized by Renilla luciferase activity. Data were analyzed by T-test. **b** The mRNA level of *FSCN1* in T24 and RT4 cells was measured by quantitative PCR (qPCR) after *miR-200b* was silenced or overexpressed. Data were analyzed by T-test. **c** The protein level of E-cadherin, N-cadherin, vimentin and FSCN1 in T24 and RT4 cells was measured by western blot after *miR-200b* was silenced or overexpressed. Data were analyzed by T-test. **d** The protein level of FSCN1 in T24 cells was measured by western blot after the cells were co-transfected with ant miR-NC or ant miR-200b and siNC or siFSCN1. Data were analyzed by T-test. **e** and **f** The migration (**e**) and invasion (**f**) abilities of T24 and RT4 cells were detected in transwell assays (without or with Matrigel) after *miR-200b* was silenced or overexpressed. **g** and **h** The cell migration (**g**) and invasion (**h**) abilities of T24 and RT4 were detected in transwell assays (without or with Matrigel) after co-transfection with miR-NC or *miR-200b* and siNC or siFSCN1. Data were analyzed by T-test. All images were taken at 100× magnification. Data are presented as the mean ± standard deviation (SD). *P < 0.05; **P < 0.01; ***P < 0.001; ns, not significant

To verify the direct binding of *miR-200b* to *FSCN1*, we performed luciferase reporter assays. We constructed a pmirGLO luciferase reporter plasmid, containing the putative (wild-type or mutated) *miR-200b* binding site sequence of *FSCN1*. We found that *miR-200b* significantly reduced the luciferase activity of the cells (T24, RT4 or 293 T) transfected with the construct containing the wild-type *FSCN1* sequence; however, *miR200b* did not have any effect on the construct with the mutated *FSCN1* sequence (Fig. 2a). Additionally, in T24 and RT4 cells, *FSCN1* was found to be downregulated by *miR-200b* and upregulated by *miR-200b* inhibitors by qPCR (Fig. 2b).

We then examined the expression of FSCN1 and EMT markers by western blot and observed that the overexpression of *miR-200b* increased the expression of E-cadherin and inhibited that of N-cadherin, vimentin and FSCN1, whereas the inhibition of *miR-200b* had opposite effect (Fig. 2c). In a rescue experiment, in T24 cells, western blot assays showed that the inhibition of *miR-200b* reversed the effect of *FSCN1* silencing (Fig. 2d). We then performed transwell assays to explore the effect of *miR-200b* on the migration and invasion abilities of BLCA cells. The overexpression of *miR-200b* in T24 and RT4 cells inhibited cell migration and invasion, whereas the inhibition of *miR-200b* had opposite effect (Fig. 2e and f). In rescue experiments in T24 and RT4 cells, the inhibition of *miR-200b* reversed the effect of *FSCN1* silencing on cell migration and invasion (Fig. 2g and h). Next, we explored the effect of *miR-200b* on cell cycle and proliferation of BLCA cells. Flow cytometry assays showed that the percentage of cells in G0/G1 phase significantly increased upon *miR-200b* overexpression and decreased upon *miR-200b* knockdown (Additional file 2: Figure S1B). Additionally, in EdU assays and RTCA on T24 and RT4 cells, we found that the proliferation of cells significantly decreased upon *miR-200b* overexpression, while *miR-200b* inhibition had the opposite effect (Additional file 2: Figure S1C and D).

These results suggest that *miR-200b* downregulates the expression of *FSCN1*, which leads to the inhibition of migration and invasion, and inhibits cell cycle progression and proliferation of BLCA cells.

### *ZEB1-AS1* is a potential regulator on the expression of *miR-200b* and *FSCN1*

**Fig. 3:**
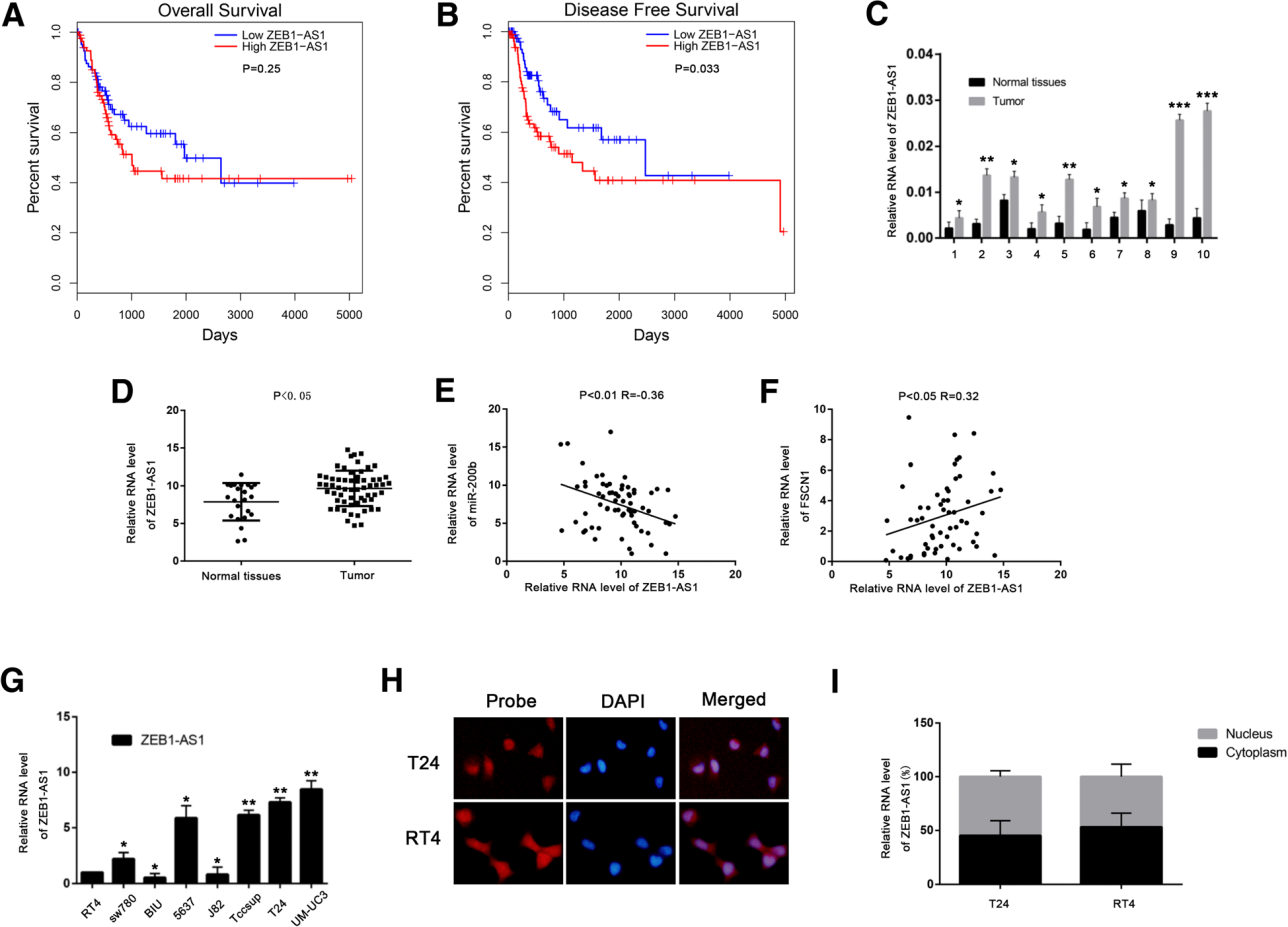
*ZEB1-AS1* is upregulated in BLCA tissues and correlates with *miR-200b* and fascin-1 (*FSCN1*). (A and B) Kaplan–Meier analysis of overall (**a**) and disease free (**b**) survival based on *ZEB1-AS1* expression levels in patients with BLCA from the TCGA database. Patients with BLCA were divided into *ZEB1-AS1* high expression group (21% of the total, with the highest *ZEB1-AS1* expression) and low expression group (21% of the total, with the lowest *ZEB1-AS1*). **c** *ZEB1-AS1* expression levels were detected in 10 pairs of BLCA and adjacent normal mucosa tissue samples by qPCR. Data were analyzed by T-test. **d** The mRNA level of *ZEB1-AS1* in 23 normal bladder tissues and 60 BLCA tissues was measured by qPCR. Data were analyzed by Mann-Whitney U test. **e** and **f** The correlation between *ZEB1-AS1* and *miR-200b* (**e**), *ZEB1-AS1* and *FSCN1* (**f**) of 60 BLCA tissues. Data were analyzed by Spearman correlation analysis. **g** The mRNA level of *ZEB1-AS1* in BLCA cell lines was measured by quantitative (qPCR). Data were analyzed by T-test. **h** Fluorescence in situ hybridization (FISH) assay in T24 and RT4 cells showing *ZEB1-AS1* (red); nuclei were stained with 4′, 6-diamidino-2-phenylindole (DAPI; blue). **i** The mRNA level of *ZEB1-AS1* in the nuclear and cytoplasmic fraction of T24 and RT4 cells by qPCR. Data are presented as mean ± standard deviation (SD). *P < 0.05; **P < 0.01; ***P < 0.001; ns, not significant

By downregulating miRNA, lncRNA can regulate their function: this is the so-called “sponge-effect.” To find potential lncRNA that could regulate *miR-200b*, we used the DIANA-LncBase tool (www.microrna.gr/LncBase) [22] and found that the lncRNA *ZEB1-AS1* might regulate the expression of *miR-200b*. We used the GEPIA server (http://gepia.cancer-pku.cn; a TCGA data portal) [23] to investigate the relation between *ZEB1-AS1* expression and the survival of patients with BLCA. We found that there was no significant correlation between *ZEB1-AS1* levels and the patient’s overall survival (Fig. 3a). On the other hand, *ZEB1-AS1* levels were positively correlated with disease free survival (Fig. 3b), indicating that high levels of *ZEB1-AS1* might result in recurrence of BLCA. As shown in Table 1, in 60 BLCA tissues, the levels of *ZEB1-AS1* were higher in high-grade tumors than in low-grade tumors. Next, we examined the correlation between *ZEB1-AS1* and *miR-200b* or *ZEB1-AS1* and *FSCN1* and found that the expression of *ZEB1-AS1* was negatively correlated with that of *miR-200b* (Additional file 2: Figure S2A) and positively correlated with that of *FSCN1* (Additional file 2: Figure S2B) according to the database.

**Table 1:** Associations between ZEB1-AS1 and clinicopathological characteristics

Parameters	Number of cases	ZEB1-AS1		*P* value
		Low	High	
Total cases	60	30	30	
Gender				
Male	45	21	24	0.371
Female	15	9	6	
Age				
≥ 60	47	22	25	0.3472
< 60	13	8	5	
Histologic grade				
Low grade	23	16	7	0.0169*
High grade	37	14	23	
TNM stage				
I,II	41	25	16	0.0125*
III,IV	19	5	14	
Lymphatic invasion				
Positive	4	1	3	0.301
Negative	56	29	27	
Distant metastasis				
Positive	0			

*P < 0.05 was considered significant

To verify this finding, we detected the expression of *ZEB1-AS1* in ten pairs of BLCA tissues and adjacent normal bladder mucosa and found significant upregulation of *ZEB1-AS1* in BLCA samples compared to normal controls (Fig. 3c). Similar results were obtained in 23 normal bladder tissues and 60 BLCA tissues (Fig. 3d). Additionally, in the BLCA samples, we found a negative correlation between *ZEB1-AS1* and *miR-200b* (Fig. 3e), and a positive correlation between *ZEB1-AS1* and *FSCN1* (Fig. 3f). We then detected the mRNA level of *ZEB1-AS1* (Fig. 3g) in BLCA cell lines and found that *ZEB1-AS1* expression was high in some high grade BLCA cell lines (Tccsup, T24, and UM-UC3), in which the expression of *miR-200b* was low, while the expression of *FSCN1* was high. To act as a sponge for miRNAs, lncRNAs need be located in the cytoplasm. Fluorescence in situ hybridization (FISH) showed that *ZEB1-AS1* was located in both the nucleus and the cytoplasm in T24 and RT4 cells (Fig. 3h). Similar results were obtained in qPCR assays in nuclear and cytoplasmic RNA fractions (Fig. 3i).

These results suggest that *ZEB1-AS1* may be a regulator on the expression of *miR-200b* and *FSCN1*.

### *ZEB1-AS1* induces migration and invasion potentially through regulating the expression of *miR-200b* and *FSCN1*

**Fig. 4:**
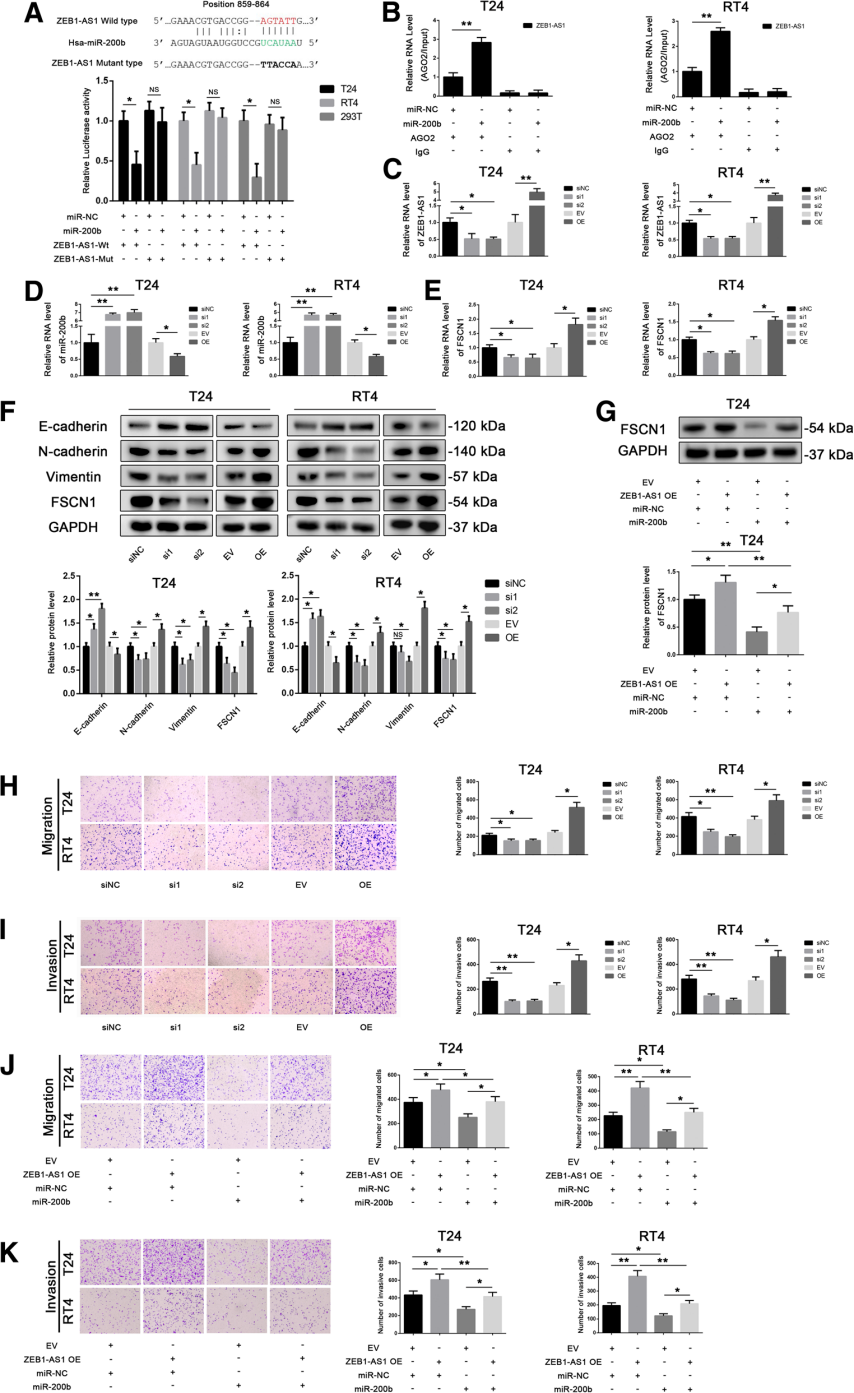
*ZEB1-AS1* downregulates *miR-200b*, upregulates fascin-1 (*FSCN1*), and promotes cell migration and invasion. **a** Luciferase assays were performed in T24, RT4, and 293 T cells co-transfected for 24 h with miR-negative control (NC) or *miR-200b* and a plasmid containing wild-type or mutant-type *ZEB1-AS1* 3′untranslated region (UTR) upstream the luciferase gene. Firefly luciferase activity of each sample was normalized by Renilla luciferase activity. Data were analyzed by T-test. **b** RNA-binding protein immunoprecipitation (RIP) assays with anti-AGO2 antibodies were performed in T24 and RT4 cells transiently transfected with *miR-200b*; *ZEB1-AS1* levels were detected by quantitative PCR (qPCR); 10% input was used as positive control and RIP with anti-IgG antibodies served as negative control. Glyceraldehyde 3-phosphate dehydrogenase (*GAPDH*) was used as the internal control. Data were analyzed by T-test. **c** Transfection efficiency of *ZEB1-AS1* knockdown (si1, si2) and overexpression (OE) in T24 and RT4 cells was detected by qPCR. Data were analyzed by T-test. **d** and **e** The levels of *miR-200b* (**d**) and *FSCN1* (**e**) were measured by qPCR after *ZEB1-AS1* knocked down or overexpressed in T24 and RT4 cells. Data were analyzed by T-test. **f** E-cadherin, N-cadherin, vimentin, and FSCN1 protein expression in T24 and RT4 cells in which *ZEB1-AS1* had been knocked down or overexpressed. Data were analyzed by T-test. (**g**) FSCN1 levels in T24 cells co-transfected with miR-NC or *miR-200b* and with an empty vector (EV) or a plasmid overexpressing *ZEB1-AS1*. Data were analyzed by T-test. **h** and **i** Transwell assays (without or with Matrigel) to detect cell migration (**h**) and invasion (**i**) of T24 and RT4 after *ZEB1-AS1* silencing or overexpression. **j** and **k** Transwell assays (without or with Matrigel) to detect cell migration (**j**) and invasion (**k**) of T24 and RT4 co-transfected with miR-NC or *miR-200b* and with an EV or a plasmid overexpressing *ZEB1-AS1*. Data were analyzed by T-test. Data are presented as the mean ± standard deviation (SD). *P < 0.05; **P < 0.01; ***P < 0.001; ns, not significant

To verify the potential interaction between *miR-200b* and *ZEB1-AS1*, we performed luciferase reporter assays. We constructed a pmirGLO luciferase reporter plasmid containing the putative *miR-200b* binding sequence (wild-type or mutated) of *ZEB1-AS1*. Luciferase assays showed that *miR-200b* significantly reduced the luciferase activity of cells (T24, RT4 or 293 T) transfected with the construct containing the wild-type *ZEB1-AS1* sequence (Fig. 4a). Additionally, RIP assays confirmed the interaction between *ZEB1-AS1* and *miR-200b* in T24 and RT4 cells through AGO2 (Fig. 4b).

Next, we transfected T24 and RT4 cells with siRNA against *ZEB1-AS1* (called si1 or si2), *ZEB1-AS1* overexpression vector (OE) and their corresponding controls; Fig. 4c shows the transfection efficiency of these constructs. The expression of *miR-200b* was decreased and that of *FSCN1* was increased after inhibiting the expression of *ZEB1-AS*1 by siRNA, while overexpression of *ZEB1-AS1* had opposite effect (Fig. 4d and e). We also evaluated the expression of FSCN1 and EMT markers by western blot. We observed that the inhibition of *ZEB1-AS1* expression increased the expression of E-cadherin and decreased the expression of N-cadherin, vimentin, and FSCN1, whereas the overexpression of *ZEB1-AS1* was associated with opposite results (Fig. 4f). In rescue experiments in T24 cells, western blot assays showed that the overexpression of *miR-200b* counteracted the overexpression of *ZEB1-AS1* (Fig. 4g).

We then performed transwell assays to explore the effect of *ZEB1-AS1* on migration and invasion of BLCA cells. Decreasing the expression of *ZEB1-AS1* by siRNA in T24 and RT4 cells inhibited cell migration and invasion, whereas overexpressing *ZEB1-AS1* had the opposite effect (Fig. 4h and i). In rescue experiments in T24 and RT4 cells, we found that the overexpression of *miR-200b* reversed the effect of the overexpression of *ZEB1-AS1* on cell migration and invasion (Fig. 4j and k). Therefore, these results suggest that *ZEB1-AS1* affects migration and invasion likely through regulating *miR-200b* and *FSCN1* in BLCA cells.

Furthermore, cellular morphology images (Additional file 2: Figure S3A) and cytoskeleton staining (Additional file 2: Figure S3B) indicated that *ZEB1-AS1* might affect the cytoskeleton of BLCA cells and therefore impact cell migration.

### *ZEB1-AS1* inhibits apoptosis, and promotes cell cycle progression and proliferation

**Fig. 5:**
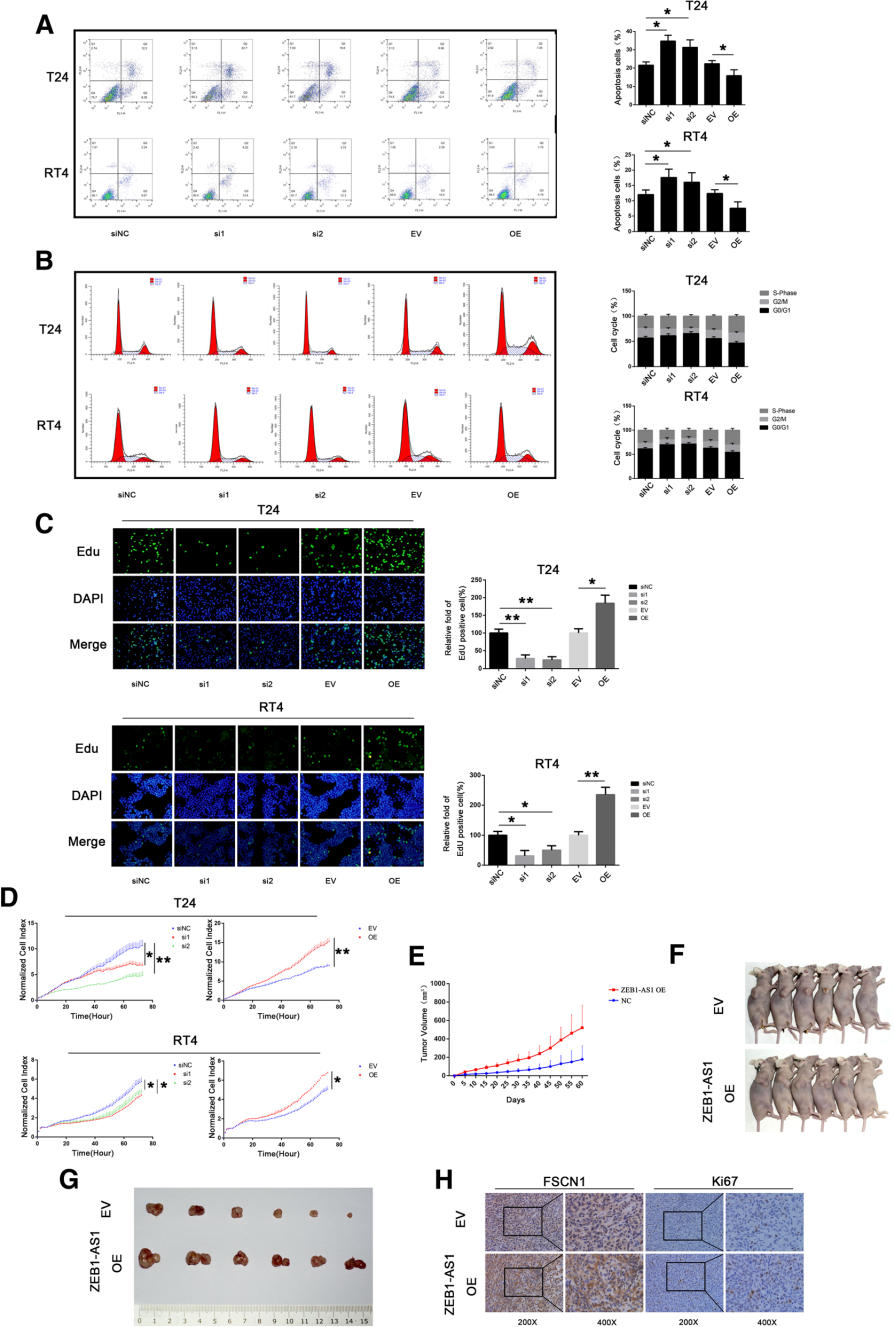
*ZEB1-AS1* inhibits apoptosis and promotes cell cycle and proliferation. **a**-**b** Apoptosis (**a**) and cell cycle (**b**) were detected by flow cytometry in T24 and RT4 cells after *ZEB1-AS1* silencing or overexpression. Data were analyzed by T-test. **c** 5-Ethynyl-2′-deoxyuridine (EdU) assays on T24 and RT4 after *ZEB1-AS1* silencing or overexpression. Data were analyzed by T-test. All images were taken at 200× magnification. **d** The proliferation of *ZEB1-AS1*-silenced or -overexpressing T24 and RT4 were detected by real time cell analysis (RTCA). Data were analyzed by T-test. **e**-**h** Xenograft model in nude mice. **e** Tumor growth was measured at the indicated days. **f** Nude mice at the end of the experiment. **g** Images of the excised tumors from each group. **h** Staining of Ki-67 and FSCN1 in the excised tumors. Data are presented as the mean ± standard deviation (SD). *P < 0.05; **P < 0.01; ***P < 0.001; ns, not significant

To further explore the function of *ZEB1-AS1* in BLCA cells, we investigated the effect of *ZEB1-AS1* on several cellular processes. Apoptosis assays in T24 and RT4 cells showed that *ZEB1-AS1* silencing was associated with a significant increase of apoptosis, and *ZEB1-*AS1 overexpression had the opposite effect (Fig. 5a). In cell cycle analysis, we found that the percentage of cells in the G0/G1 phase was increased upon *ZEB1-AS1* silencing and decreased when *ZEB1-AS1* was overexpressed (Fig. 5b). Additionally, EdU assays and RTCA showed that the proliferation of cells was decreased when *ZEB1-AS1* was silenced and increased upon *ZEB1-AS1* overexpression (Fig. 5c and d).

We extended this result in xenograft models. First, we generated UM-UC3 cells stably overexpressing *ZEB1-AS1*. Then, we injected mice with these cells (or control UM-UC3) and observed the formation of tumors. The tumors derived from *ZEB1-AS1* overexpressing cells grew faster than control cells (Fig. 5e-g). The IHC assay showed a high expression level of Ki67 and FSCN1 in tumors derived from *ZEB1-AS1* overexpressing cells (Fig. 5h).

These results suggest that *ZEB1-AS1* inhibits apoptosis and enhances tumor growth both in vitro and in vivo.

### *ZEB1-AS1* enhances cell migration and invasion that is induced by TGF-β1

**Fig. 6:**
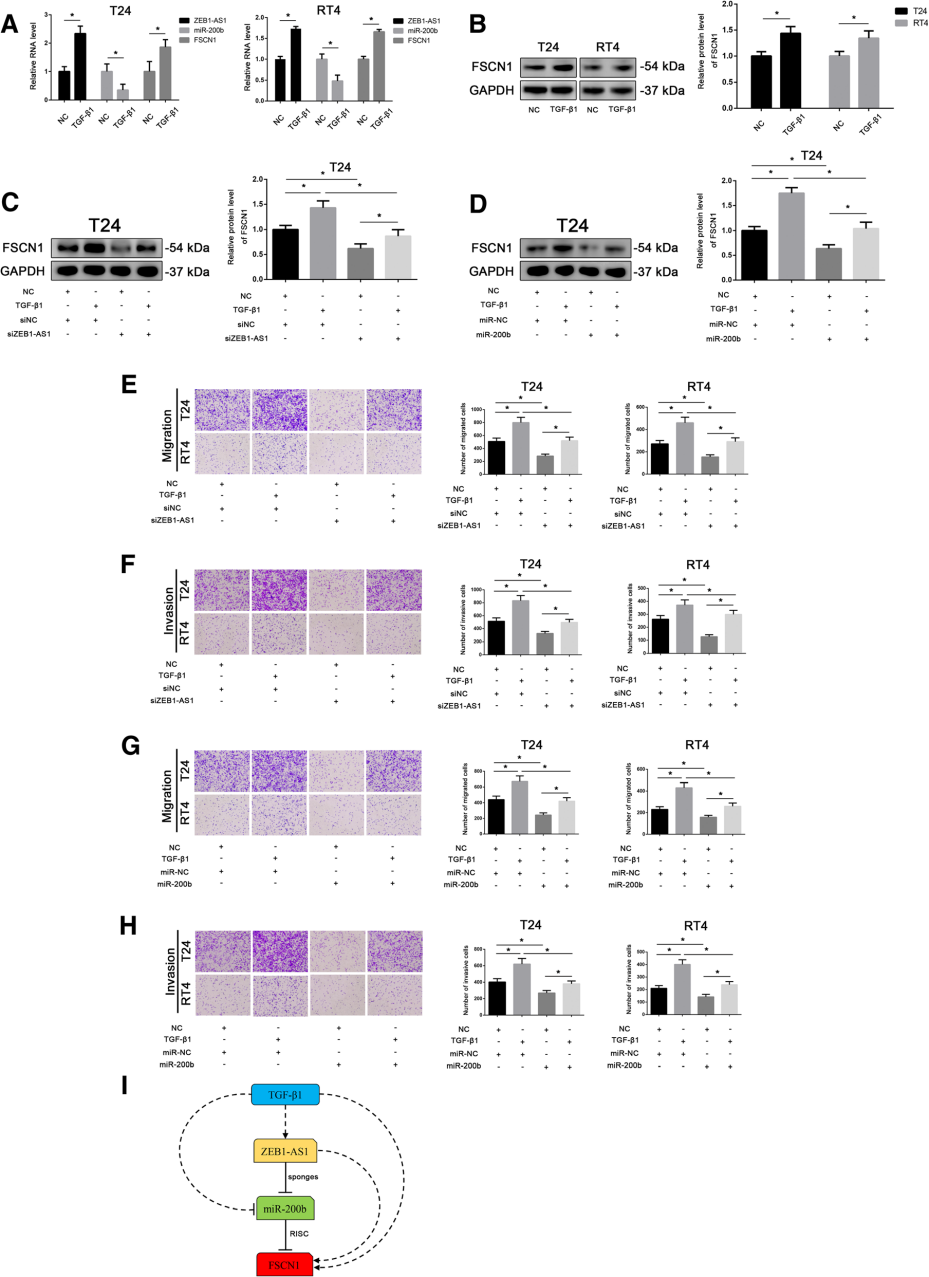
Transforming growth factor (TGF)-β1 inhibits *miR-200b* and promotes *ZEB1-AS1* and fascin-1 (FSCN1) expression. **a** The mRNA level of *ZEB1-AS1*, *miR-200b* and *FSCN1* in T24 and RT4 after treated with TGF-β1. Data were analyzed by T-test. **b** FSCN1 protein expression in T24 and RT4 cells treated with TGF-β1. Data were analyzed by T-test. **c** FSCN1 protein expression in T24 cells treated with TGF-β1 and transfected with siNC or si*ZEB1-AS1*; untreated and not-transfected cells were used as control. Data were analyzed by T-test. **d** FSCN1 protein levels in T24 cells treated with TGF-β1 and transfected with miR-negative control (miR-NC) or *miR-200b*; untreated and not-transfected cells were used as control. Data were analyzed by T-test. **e** and **f** Transwell assays (without or with Matrigel) to detect migration (**e**) and invasion (**f**) of T24 and RT4 treated with TGF-β1 and transfected with siNC or siZEB1-AS1; untreated and not-transfected cells were used as control. **g** and **h** Transwell assays (without or with Matrigel) to detect the migration (**g**) and invasion (**h**) of T24 and RT4 treated with TGF-β1 and transfected with miR-NC or *miR-200b* untreated and not-transfected cells were used as control. Data were analyzed by T-test. **i** Hypothetical model illustrating the TGF-β1/*ZEB1-AS1*/*miR-200b*/FSCN1 axis. Data are presented as the mean ± standard deviation (SD). *P < 0.05; **P < 0.01; ***P < 0.001; ns, not significant

It is well known that TGF-β1 induces cell migration and invasion and downregulates the expression of *miR-200b* in many kinds of cancers. In our previous studies, we found that TGF-β1 induced cell migration and invasion by upregulating the expression of FSCN1 in BLCA and kidney renal clear cell carcinoma (KIRC). Therefore, we asked whether TGF-β1 regulated the *ZEB1-AS1*/*miR-200b/*FSCN1 axis. Interestingly, we found that TGF-β1 treatment in T24 and RT4 cells was associated with upregulation of *ZEB1-AS1* and *FSCN1* and downregulation of *miR-200b* (Fig. 6a and b).

To further verify this observation, we performed rescue experiments. Western blot assays showed that *ZEB1-AS1* silencing reversed the upregulation of FSCN1 induced by TGF-β1 treatment in T24 cells (Fig. 6c). At the same time, *miR-200b* overexpression had a similar effect (Fig. 6d) on the regulation of FSCN1 by TGF-β1. Similar results were obtained in cell migration (Fig. 6e and g) and invasion (Fig. 6f and h) assays in T24 and RT4 cells.

Therefore, we concluded that, in BLCA, *ZEB1-AS1* is likely to be involved in TGF-β1 regulation on cell migration and invasion potentially through impacting the expression of *miR-200b* and FSCN1 (Fig. 6i).

## Discussion

In our previous study, we found that FSCN1 is a biomarker of BLCA [12, 13]. In this study, we investigated the regulatory mechanisms through which FSCN1 exerts its effect in BLCA.

miRNAs are non-coding RNA that regulate the expression of genes through the RNA-induced silencing complex (RISC) [24, 25]. Through bioinformatics analysis, we found that *miR-200b* might regulate the expression of *FSCN1* and verified this hypothesis by performing experiments on BLCA cells. Even though the expression of *miR-200b* in BLCA samples and normal tissues was not significantly different probably due to the small sample size in our study, *miR-200b* was still correlated with good prognosis (overall survival).

*miR-200b* inhibits cell migration and invasion, negatively affects cell cycle progression and inhibits cell proliferation, and is therefore considered a tumor suppressor in BLCA. *miR-200b* exerts a similar function in other types of cancer [26, 27]. Some studies showed that the levels of *miR-200* family were increased in BLCA samples compared with normal tissues but decreased in high grade and stage tumors in both non-muscle invasive bladder cancer (NMIBC) and muscle invasive bladder cancer (MIBC) compared with low grade and stage tumor, still acting as a tumor suppressor [14, 28–30]. These observations are in agreement with our results. How *miR-200b* causes these effects in BLCA needs to be further investigated.

The “sponge effect” of lncRNAs is the classic mechanism through which they regulate miRNAs [31–34]. Bioinformatics analysis indicated that *ZEB1-AS1* might regulate the function of *miR-200b*. *ZEB1-AS1* is an oncogenic lncRNA correlated with higher histopathological grade in many kinds of cancer [35–38], and recent studies have suggested that *ZEB1-AS1* regulates the *miR-200c* in prostate cancer [37], and *miR-200* family in osteosarcoma [39]. However, whether there is a regulatory relation between *ZEB1-AS1* and *miR-200b* in BLCA is still unknown.

Our bioinformatics analysis suggested *ZEB1-AS1* is more expressed in BLCA tissues than in normal ones and correlates with disease-free survival and not with overall survival in patients with BLCA. Therefore, *ZEB1-AS1* might promote tumor progression by increasing the recurrence of BLCA. Further experiments demonstrated that *ZEB1-AS1* acted as a competing endogenous RNA (ceRNA) in BLCA by interacting with *miR-200b* and indirectly upregulating *FSCN1*. Furthermore, in functional experiments, we found that *ZEB1-AS1* decreased cell apoptosis and G0/G1 arrest and induced BLCA cell proliferation, migration, and invasion, thereby exerting a tumor promoting role via the *miR-200b*/FSCN1 axis.

TGF-β1 affects many biological processes [16] such as migration, invasion, and proliferation. In our previous studies, we found that TGF-β1 upregulated the expression of FSCN1 in BLCA [19] and KIRC [40]. Some studies have shown that TGF-β1 downregulates the expression of *miR-200b* [18, 41, 42]. Therefore, we investigated if, in our system, TGF-β1 acted through the *ZEB1-AS1*/*miR-200b*/FSCN1 axis and found that TGF-β1 upregulated the expression of *ZEB1-AS1*. These results suggest that TGF-β1 induces migration and invasion in BLCA by regulating the *ZEB1-AS1*/*miR-200b*/FSCN1 axis.

In the current study, we focused on the effect of the *ZEB1-AS1*/*miR-200b*/FSCN1 axis on cell migration and invasion. This axis might affect other cell functions; however, their mechanism has not been investigated in this study and might be the focus of future research.

Some studies have shown that TGF-β1 increases FSCN1 expression via the extracellular-signal-regulated kinase (ERK) and c-Jun N-terminal kinase (JNK) signaling pathways [40, 43]. Meanwhile, some studies reported that *ZEB1-AS1* could affect cell function by regulating the Wnt/β-catenin [44] and p38MAPK signaling pathways, [45] which were also regulated by TGF-β1 [46–49]. Therefore, we hypothesize that some signaling network must participate in the regulation of the TGF-β1/*ZEB1-AS1*/*miR-200b*/FSCN1 axis. Future studies might investigate the conditions or cases in which the axes are activated.

## Conclusions

Our study suggests that the lncRNA *ZEB1-AS1* is upregulated in BLCA, correlates with high tumor grade and high TNM stage and indicates poor prognosis of patients. Functional experiments showed that *ZEB1-AS1* induces cell migration, invasion, and proliferation in BLCA. TGF-β1 might induce migration and invasion, at least in part, by regulating the *ZEB1-AS1*/*miR-200b*/FSCN1 axis in BLCA. Thus, the TGF-β1/*ZEB1-AS1*/*miR-200b*/FSCN1 axis may be a potential therapeutic target for BLCA.

## Additional files

Additional file 1: **Table S1.** Sequences of RNAi for transfection. **Table S2.** Sequences of primer pairs for qPCR. **Table S3.** Sequences of ZEB1-AS1 probe for FISH. (DOCX 13 kb)

Additional file 2: **Figure S1.** miR-200b is correlated with FSCN1 and inhibits cell cycle and proliferation (A) Correlation between FSCN1 and miR-200b in patients with BLCA from the TCGA database. Data were analyzed by Spearman correlation analysis. (B) The cell cycle of T24 and RT4 cells was detected by flow cytometry after miR-200b was silenced or overexpressed. Data were analyzed by T-test. (C) 5-Ethynyl-2′-deoxyuridine (EdU) assays were performed on T24 and RT4 cells after miR-200b was silenced or overexpressed. Data were analyzed by T-test. All images were taken at 200× magnification. (D) The proliferation of T24 and RT4 cells was detected by real time cell analysis (RTCA) for 72 h after miR-200b was silenced or overexpressed. Data were analyzed by T-test. Data are presented as the mean ± standard deviation (SD). *P < 0.05; **P < 0.01; ***P < 0.001; ns, not significant. **Figure S2.** ZEB1-AS1 is correlated with miR-200b and FSCN1 according to TCGA database (A and B) Correlation between ZEB1-AS1 and miR-200b (A) and ZEB1-AS1 and FSCN1 (B) in patients with BLCA from the TCGA database. Data were analyzed by Spearman correlation analysis. **Figure S3.** The cellular morphological changes after ZEB1-AS1 has been overexpressed. (A) Cellular morphology images of T24 and RT4 cells in which ZEB1-AS1 had been overexpressed. (B) Cytoskeleton staining images of T24 cells in which ZEB1-AS1 had been overexpressed. (TIF 3996 kb)

## Abbreviations

BLCA: Bladder cancer
ceRNA: Competing endogenous RNA
DAPI: 4′, 6-diamidino-2-phenylindole
EDTA: Ethylenediaminetetraacetic acid
EdU: 5-Ethynyl-2′- deoxyuridine
EMT: Epithelial-to-mesenchymal transition
ERK: Extracellular-signal-regulated kinase
EV: Empty vector
FBS: Fetal bovine serum
FISH: Fluorescence in situ hybridization
FITC: Fluorescein isothiocyanate
FSCN1: Fascin-1
GAPDH: Glyceraldehyde 3-phosphate dehydrogenase
IHC: Immunohistochemistry
JNK: c-Jun N-terminal kinase
lncRNA: Long non-coding RNA
MIBC: Muscle invasive bladder cancer
miRNA: microRNA
NC: Negative control
NMIBC: Non-muscle invasive bladder cancer
OE: Overexpression vector
PBS: Phosphate-buffered saline
PI: Propidium iodide
qPCR: Quantitative PCR
RIP: RNA-binding Protein Immunoprecipitation
RISC: RNA-induced silencing complex
RTCA: Real time cell analysis
SD: Standard deviation
siRNA: Short interfering RNA
TBS-T: Tris-buffered saline with 1% Tween 20
TCGA: The Cancer Genome Atlas
TGF: Transforming growth factor
UTR: Untranslated region
ZEB1: Zinc Finger E-Box Binding Homeobox 1
