# Supplementary material for: Long non-coding RNA ZEB1-AS1 regulates miR-200b/FSCN1 signaling and enhances migration and invasion induced by TGF-β1 in bladder cancer cells
Source: J Exp Clin Cancer Res. 2019 Mar 1;38:111. doi: 10.1186/s13046-019-1102-6 (PMC6397446; doi:10.1186/s13046-019-1102-6)
Supplement: Supplementary file 1 — Table S1. Sequences of RNAi for transfection. Table S2. Sequences of primer pairs for qPCR. Table S3. Sequences of ZEB1-AS1 probe for FISH. (DOCX 13 kb) [file 13046_2019_1102_MOESM1_ESM.docx]

**Table S1**

| Sequences of RNAi for transfection | |
| --- | --- |
| Gene | Nucleotide sequence(5'-3') |
| siNC | UUCUCCGAACGUGUCACGUTT |
|  | ACGUGACACGUUCGGAGAATT |
| siZEB1-AS1-1 | CUUCAAUGAGAUUGAACUUCA |
|  | AAGUUCAAUCUCAUUGAAGUC |
| siZEB1-AS1-2 | GAAAGAGAGGCUAGAAGUUCC |
|  | AACUUCUAGCCUCUCUUUCAA |
| siFSCN1-1 | GCUGCUACUUUGACAUCGATT |
|  | UCGAUGUCAAAGUAGCAGCTT |
| siFSCN1-2 | GCGCCUACAACAUCAAAGATT |
|  | UCUUUGAUGUUGUAGGCGCTT |
| miR-NC | UUCUCCGAACGUGUCACGUTT |
|  | ACGUGACACGUUCGGAGAATT |
| miR-200b | UAAUACUGCCUGGUAAUGAUGA |
|  | AUCAUUACCAGGCAGUAUUAUU |
| ant miR-NC | CAGUACUUUUGUGUAGUACAA |
| ant miR-200b | UCAUCAUUACCAGGCAGUAUUA |

**Table S2**

| Sequences of primer pairs for qPCR | |
| --- | --- |
| Gene | Nucleotide sequence(5'-3') |
| FSCN1 | F:CTGCTACTTTGACATCGAGTGG |
|  | R:GGGCGGTTGATGAGCTTCA |
| ZEB1-AS1 | F:GAGGCTAGAAGTTCCGCTTG |
|  | R:CGGATGGGGAAGTGAGACAA |
| GAPDH | F:ACAACTTTGGTATCGTGGAAGG |
|  | R:GCCATCACGCCACAGTTTC |
| miR-200b-3p | F:GGTAATACTGCCTGGTAATGATG |
|  | R:Provided by Mir-X™ miRNA First Strand Synthesis Kit(Clonetech,Code No.638315) |
| U6 | F:Provided by Mir-X™ miRNA First Strand Synthesis Kit(Clonetech,Code No.638315) |
|  | R:Provided by Mir-X™ miRNA First Strand Synthesis Kit(Clonetech,Code No.638315) |
| F, forward; R, reverse | |

**Table S3**

| Sequences of ZEB1-AS1 probe for FISH | |
| --- | --- |
|  | Nucleotide sequence(5'-3') |
| 1 | CGTG+TGGGTATTAC+TCATCC |
| 2 | TAGG+AAGGAATTCA+TGGCCT |
| 3 | GGCCC+AAACTAAC+TAAACCA |
| 4 | CCTTAC+TGTCAAGAAC+AGGG |
