# Supplementary figures and images for: Long non-coding RNA ZEB1-AS1 regulates miR-200b/FSCN1 signaling and enhances migration and invasion induced by TGF-β1 in bladder cancer cells
Source: J Exp Clin Cancer Res. 2019 Mar 1;38:111. doi: 10.1186/s13046-019-1102-6 (PMC6397446; doi:10.1186/s13046-019-1102-6)

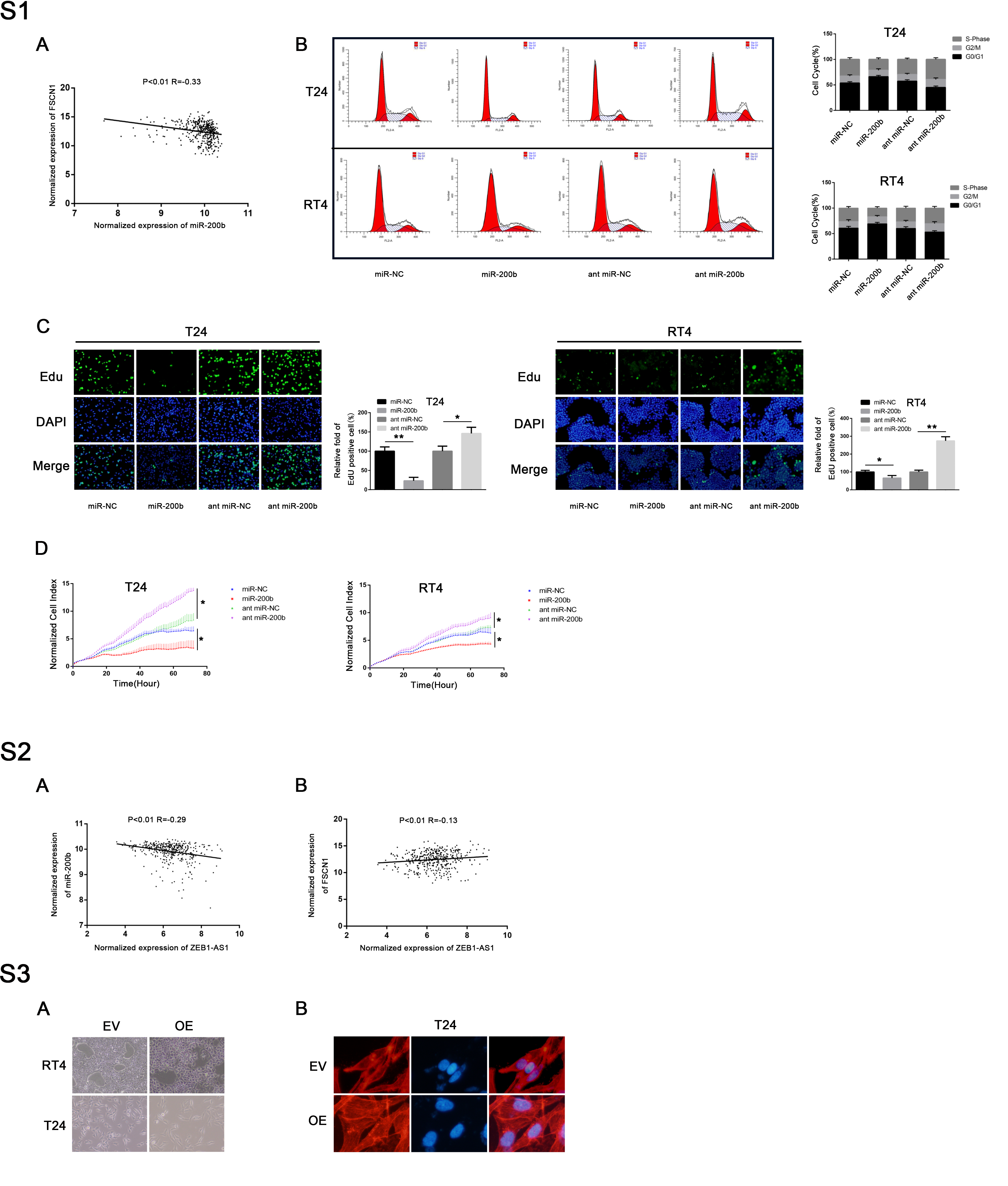

Supplement: Supplementary file 2 — Figure S1. miR-200b is correlated with FSCN1 and inhibits cell cycle and proliferation (A) Correlation between FSCN1 and miR-200b in patients with BLCA from the TCGA database. Data were analyzed by Spearman correlation analysis. (B) The cell cycle of T24 and RT4 cells was detected by flow cytometry after miR-200b was silenced or overexpressed. Data were analyzed by T-test. (C) 5-Ethynyl-2′-deoxyuridine (EdU) assays were performed on T24 and RT4 cells after miR-200b was silenced or overexpressed. Data were analyzed by T-test. All images were taken at 200× magnification. (D) The proliferation of T24 and RT4 cells was detected by real time cell analysis (RTCA) for 72 h after miR-200b was silenced or overexpressed. Data were analyzed by T-test. Data are presented as the mean ± standard deviation (SD). *P < 0.05; **P < 0.01; ***P < 0.001; ns, not significant. Figure S2. ZEB1-AS1 is correlated with miR-200b and FSCN1 according to TCGA database (A and B) Correlation between ZEB1-AS1 and miR-200b (A) and ZEB1-AS1 and FSCN1 (B) in patients with BLCA from the TCGA database. Data were analyzed by Spearman correlation analysis. Figure S3. The cellular morphological changes after ZEB1-AS1 has been overexpressed. (A) Cellular morphology images of T24 and RT4 cells in which ZEB1-AS1 had been overexpressed. (B) Cytoskeleton staining images of T24 cells in which ZEB1-AS1 had been overexpressed. (TIF 3996 kb) [file 13046_2019_1102_MOESM2_ESM.tif]
